# Supplementary material for: The Boltzmann fair division for distributive justice
Source: Sci Rep. 2022 Sep 28;12:16179. doi: 10.1038/s41598-022-19792-3 (PMC9519911; doi:10.1038/s41598-022-19792-3)
Supplement: Supplementary file 1 — Supplementary Information. [file 41598_2022_19792_MOESM1_ESM.docx]

**Supplementary Information for**

The Boltzmann fair division for distributive justice

**Ji-Won Park^1,2^*, Jaeup U. Kim^3^, Cheol-Min Ghim^3^, Chae Un Kim^3^***

^1^Regional Science, Cornell University, Ithaca, NY 14853, USA

^2^Department of Economics, University of Ulsan, Ulsan, 44610, Korea

^3^Department of Physics, Ulsan National Institute of Science and Technology (UNIST), Ulsan 44919, Korea

* **Corresponding authors: Ji-Won Park and Chae Un Kim**.

Email: jp429@cornell.edu

Email: cukim@unist.ac.kr

**Existence of finite *β* value maximizing the total utility function in the homogeneous Boltzmann division**

As long as the utility function for each player is upper-bounded, the total utility is a decreasing function at a large enough *β* where the player with the highest contribution receives most of the cake units. This observation indicates that the total utility function is maximized at a nonzero *β* if it is an increasing function near *β* = 0. To clarify this condition, let us first analyze the behavior of the utility function at a small *β* regime. Using Taylor expansion, the Boltzmann probability for the homogenous cake-cutting (Eq. 1 in Methods) can be approximated as

$P_{j}=\frac{1+\beta E_{j}}{n+\sum_{k=1}^{n} \beta E_{k}}+O\left(\beta^{2}\right)$, for *j =* 1,2, ∙∙∙, *n* (S1)

Then, the number of cake units allocated to each player is further approximated as

$N_{j}=\frac{\tilde{N}}{n}\left(1+\beta E_{j}-\beta\bar{E}\right)+O\left(\beta^{2}\right)$, for *j =* 1,2, ∙∙∙, *n* (S2)

where $\bar{E}\equiv\sum_{k=1}^{n} E_{k}/n$.

Now, let us consider the behavior of a utility function $u_{j}\left(x\right)$ where *x* is the number of cake units allocated to player *j*. Note that the utility function $u_{j}\left(x\right)$ here is a nonlinear function (not necessarily a hyperbolic tangent) that reflects the realistic utility of the participating players. At *β* = 0, each player receives the same amount of cake units, $x=\tilde{N}/n$, and thus it is helpful to apply the Taylor expansion near this uniform division *x* value,

$u_{j}\left(x\right)=A_{j}+C_{j}\left(x-\frac{\tilde{N}}{n}\right)+O\left(\left(x-\frac{\tilde{N}}{n}\right)^{2}\right)$ (S3)

where $A_{j}$ and $C_{j}$ are the value and slope of $u_{j}\left(x\right)$ at $x=\tilde{N}/n$, respectively.

Using Eq. S2, the utility function of player *j* and the total utility function (*U*) up to the first order in *β* is given by

$u_{j}\left(N_{j}\right)\cong A_{j}+\frac{\tilde{N}\beta}{n}\left(C_{j}E_{j}-C_{j}\bar{E}\right), U=\sum_{j=1}^{n} u_{j}\left(N_{j}\right)\cong U_{0}+\tilde{N}\beta\left(\frac{1}{n}\sum_{j=1}^{n} C_{j}E_{j}-\bar{C}\bar{E}\right)$ (S4)

where $U_{0}\equiv\sum_{j=1}^{n} A_{j}$ and $\bar{C}\equiv\sum_{j=1}^{n} C_{j}/n$.

This result indicates that the total utility (*U*) becomes an increasing function of *β* and that its maximization at nonzero *β* is guaranteed when the following condition is satisfied.

$\frac{1}{n}\sum_{j=1}^{n} C_{j}E_{j}>\bar{C}\bar{E}$ (S5)

Such a condition (Eq. S5) is not always satisfied for an arbitrary form of utility function. In this paper, we used a hyperbolic tangent-shaped utility function whose saturation maximum value is set at 1 for each player (Fig. S1). In this case, if the cake is large enough relative to the players’ needs so that the individual utility function is nearly saturated for some players, the condition (Eq. S5) can be satisfied when the players with higher contributions have higher needs. On the other hand, if the cake is small relative to the players’ needs so that the individual utility is far from 1 for every player, the condition (Eq. S5) can be satisfied when the players with higher contributions have smaller needs.

For many realistic fair-division problems, the utility functions do not need to have the same saturation maximum for all players, and it might be possible that the heavy contributor’s utility function has the tendency to have a higher slope ($C_{j}$) regardless of the number of allocated cake units. If the individual utility function of a heavy contributor has a higher slope and higher saturation maximum value (Fig. S2), then there will always exist a nonzero *β* value maximizing the total utility.

Introducing heterogeneity to the preference of cake flavor drastically complicates the problem, and each player’s share is no longer equal to $\tilde{N}/n$ at *β* = 0. A simple analysis linearizing the utility function is not available, but the short conclusion mentioned above is still valid to a degree.


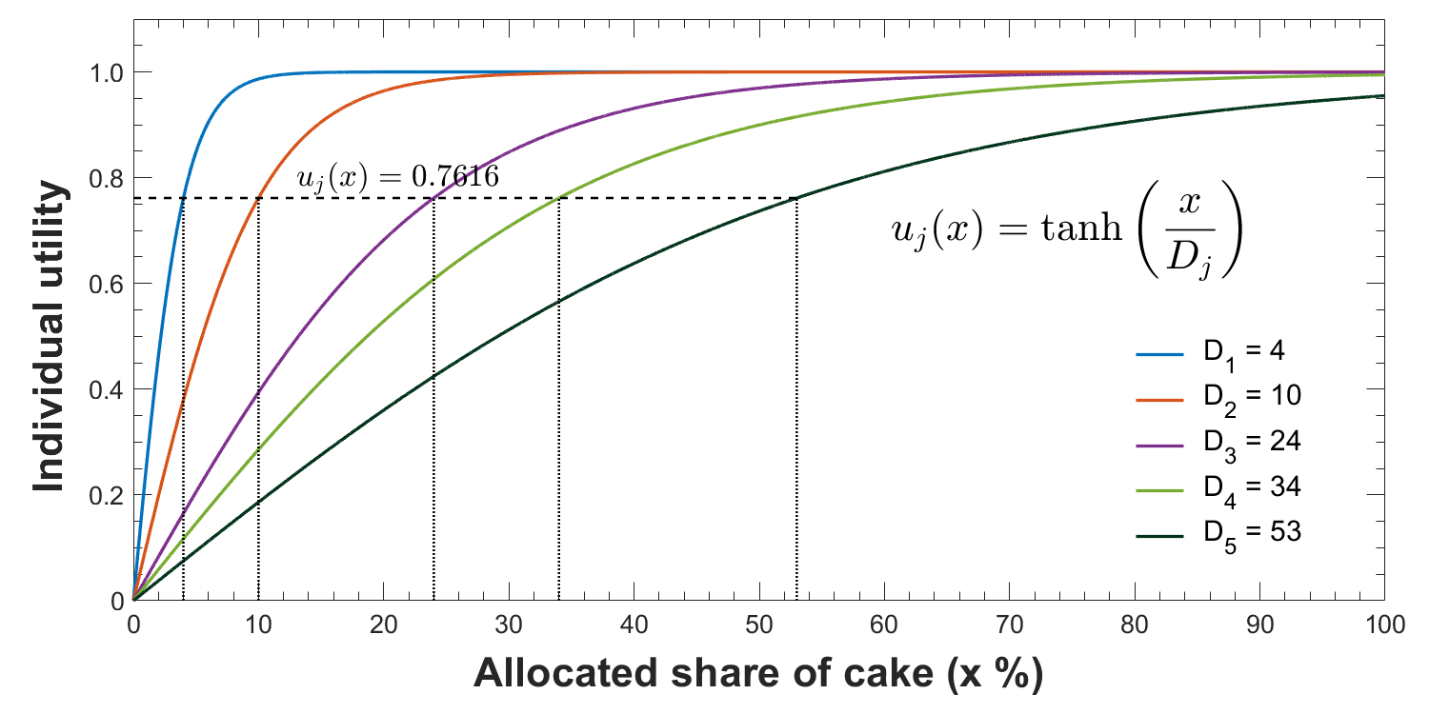


**Fig. S1.** Individual utility function for the five participating players. It was assumed that the players’ needs are 4%, 10%, 24%, 34%, and 53%, respectively. Note that when the share of cake is the same as the players’ needs, the utility function becomes saturated with the value, *u_j_* (*D_j_*) = 0.7616.


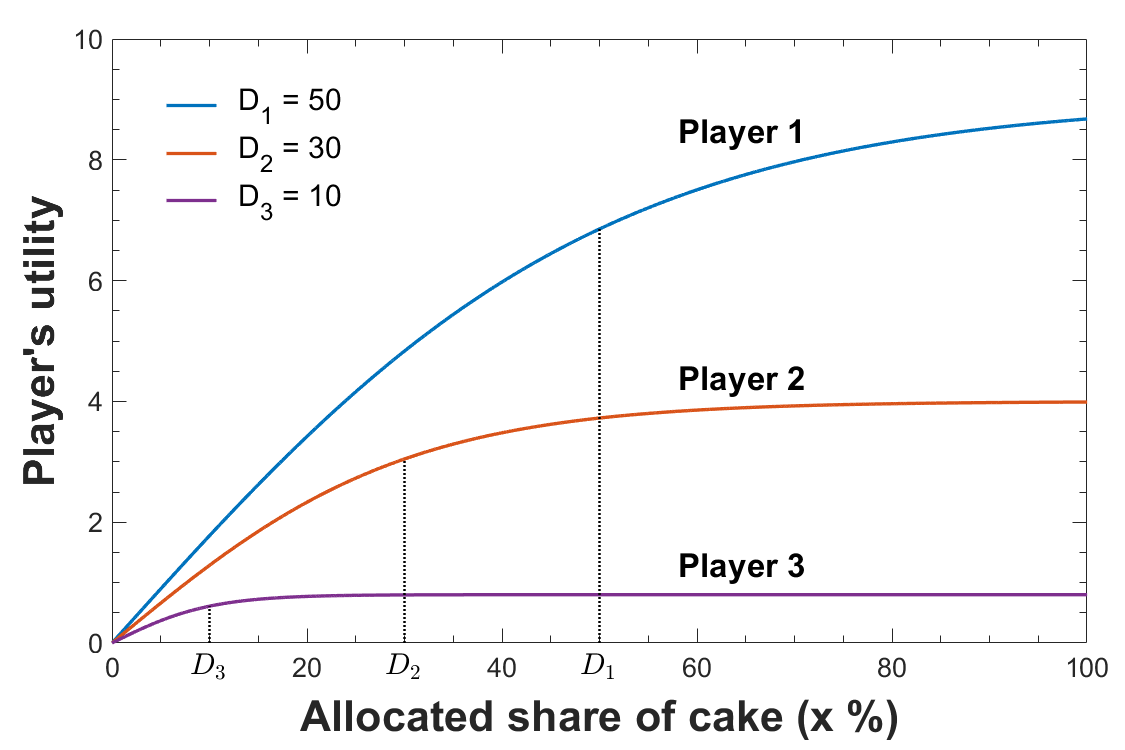


**Fig. S2.** Utility function for the three participating players. The saturation level of the hyperbolic tangent functions is set at 9, 4, and 0.8, respectively. As the division potential (*E_j_*) reflecting player *j*’s contribution increases (*E*_1_>*E*_2_>*E*_3_), the player’s utility function has a higher slope at any allocated share of cake, thus leading to higher saturation values. In this case, the total utility (*U*) can always be maximized at a nonzero *β* value. This situation would be suitable when the participating players represent a group of individuals, or communities and nations, that may have distinct population sizes and/or value systems.

Table S1. Contributions, needs, and weight factors of five participating players.

| **Player** | **Contributions** | **Needs** | **Weight factor (**$w_{j}^{i}$**)**  **(in heterogeneous cake-cutting)** | | | |
| --- | --- | --- | --- | --- | --- | --- |
|  | $E_{j}$ | $D_{j}$ | **Vanilla** | **Chocolate** | **Strawberry** | **Broccoli** |
| **1** | 5 | 4 | 0.25 | 0.25 | 0.25 | 0.25 |
| **2** | 10 | 10 | 0.5 | 0.25 | 0.25 | 0 |
| **3** | 20 | 24 | 0 | 1 | 0 | 0 |
| **4** | 25 | 34 | 0.5 | 0 | 0 | 0.5 |
| **5** | 40 | 53 | 0.25 | 0.25 | 0.5 | 0 |

Note that the constraint $\sum_{i=1}^{4} w_{j}^{i}=1$ should be met for each player *j.*

Table S2. Homogeneous cake-cutting using the Boltzmann division ($\beta^{*}=0.0288$)

| **Player** | ***E_j_*** | ***D_j_*** | $e^{\beta^{*}E_{j}}$ | $P_{j}$ | $N_{j}$ |
| --- | --- | --- | --- | --- | --- |
| **1** | 5 | 4 | 1.15 | 0.12 | 12.17 |
| **2** | 10 | 10 | 1.33 | 0.14 | 14.06 |
| **3** | 20 | 24 | 1.78 | 0.19 | 18.75 |
| **4** | 25 | 34 | 2.05 | 0.22 | 21.66 |
| **5** | 40 | 53 | 3.16 | 0.33 | 33.36 |
| **Sum** | **100** | **125** | **9.49** | **1.00** | **100** |

Table S3. Heterogeneous cake-cutting using the Boltzmann division ($\beta^{*}=0.0286$)

| **Player** | $w_{j}^{i}e^{\beta^{*}E_{j}}$ | | | | $P_{j}^{i}=\frac{w_{j}^{i}e^{\beta^{*}E_{j}}}{\sum_{k=1}^{n} \left(w_{k}^{i}e^{\beta^{*}E_{k}}\right)}$ | | | | $N_{j}^{i}$ | | | | | $N_{j}$ |
| --- | --- | --- | --- | --- | --- | --- | --- | --- | --- | --- | --- | --- | --- | --- |
|  | **V** | **C** | **S** | **B** | **V** | **C** | **S** | **B** | **V** | **C** | **S** | **B** | **Total** | |
| **1** | 0.29 | 0.29 | 0.29 | 0.29 | 0.10 | 0.09 | 0.13 | 0.22 | 2.61 | 2.27 | 3.29 | 5.50 | 13.67 | |
| **2** | 0.67 | 0.33 | 0.33 | 0.00 | 0.24 | 0.10 | 0.15 | 0.00 | 6.03 | 2.62 | 3.80 | 0.00 | 12.44 | |
| **3** | 0.00 | 1.77 | 0.00 | 0.00 | 0.00 | 0.56 | 0.00 | 0.00 | 0.00 | 13.94 | 0.00 | 0.00 | 13.94 | |
| **4** | 1.02 | 0.00 | 0.00 | 1.02 | 0.37 | 0.00 | 0.00 | 0.78 | 9.26 | 0.00 | 0.00 | 19.50 | 28.75 | |
| **5** | 0.78 | 0.78 | 1.57 | 0.00 | 0.28 | 0.25 | 0.72 | 0.00 | 7.11 | 6.17 | 17.91 | 0.00 | 31.19 | |
| **Sum** | **2.76** | **3.18** | **2.19** | **1.31** | **1.00** | **1.00** | **1.00** | **1.00** | **25.00** | **25.00** | **25.00** | **25.00** | **100.00** | |

V=Vanilla, C=Chocolate, S=Strawberry, B=Broccoli
